# Higher Serum Concentrations of N-Terminal Pro-B-Type Natriuretic Peptide Associate with Prevalent Hypertension whereas Lower Associate with Incident Hypertension

**DOI:** 10.1371/journal.pone.0117864

**Published:** 2015-02-06

**Authors:** Ekim Seven, Lise L. N. Husemoen, Hans Ibsen, Nele Friedrich, Matthias Nauck, Kristian Wachtell, Allan Linneberg, Jørgen L. Jeppesen

**Affiliations:** 1 Department of Internal Medicine, Glostrup Hospital, University of Copenhagen, Glostrup, Denmark; 2 Research Centre for Prevention and Health, the Capital Region of Denmark, Glostrup, Denmark; 3 Department of Internal Medicine, Holbæk Hospital, University of Copenhagen, Holbæk, Denmark; 4 Faculty of Health and Medical Sciences, University of Copenhagen, Copenhagen, Denmark; 5 Institute of Clinical Chemistry and Laboratory Medicine, University Medicine Greifswald, Greifswald, Germany; 6 Department of Clinical Experimental Research, Glostrup Hospital, Glostrup, Denmark; Bielefeld Evangelical Hospital, GERMANY

## Abstract

**Background:**

The role of the natriuretic peptides (NPs) in hypertension is complex. Thus, a higher blood NP concentration is a robust marker of pressure-induced cardiac damage in patients with hypertension, whereas genetically elevated NP concentrations are associated with a reduced risk of hypertension and overweight individuals presumably at high risk of hypertension have lower NP concentrations.

**Objective:**

To investigate the associations between serum N-terminal pro-B-type natriuretic peptide (NT-proBNP), used as a surrogate marker for active BNP, and prevalent as well as 5-year incident hypertension in a Danish general population sample.

**Methods:**

Cross-sectional and prospective population-based study.

**Results:**

At baseline, among 5,307 participants (51.3% women, mean age 46.0±7.9 years) with a complete set of data, we recorded 1,979 cases with prevalent hypertension (PHT). Among 2,389 normotensive participants at baseline with a complete set of data, we recorded 324 cases with incident hypertension (IHT) on follow-up 5 years later. In models adjusted for age, sex, lifestyle, social, dietary, anthropometric, pulmonic, lipid, metabolic and renal risk factors, as well as heart rate and baseline blood pressure (only incident model), one standard deviation increase in baseline log-transformed NT-proBNP concentrations was on one side associated with a 21% higher risk of PHT (odds ratio [OR]: 1.21 [95% confidence interval (CI): 1.13-1.30], *P<0.001*), and on the other side with a 14% lower risk of IHT (OR: 0.86 [95%CI:0.76-0.98], *P* = *0.020*).

**Conclusions:**

Higher serum concentrations of NT-proBNP associate with PHT whereas lower concentrations associate with IHT. This suggests that a lower amount of circulating BNP, resulting in diminished vasodilation and natriuresis, could be involved in the pathogenesis of hypertension in its early stages.

## Introduction

A higher serum concentration of B-type natriuretic peptide (BNP) is a robust marker of pressure-induced cardiac damage, e.g. left atrial enlargement and left ventricular hypertrophy, in patients with hypertension [1]. In contrast, in cross-sectional Mendelian randomization studies, genetically elevated serum concentrations of BNP or the N-terminal fragment of proBNP (NT-proBNP) have been shown to associate with a reduced risk of hypertension [2,3]. This suggests that a complex relationship exists between BNP and hypertension, and to complicate things further, it has recently been shown that obese patients with newly diagnosed uncomplicated hypertension have lower than expected serum concentrations of BNP taking their high salt intake and high blood pressure into account [4]. Thus, a lower amount of circulating BNP, resulting in diminished vasodilation and natriuresis, could be involved in the pathogenesis of hypertension in its early stages [5,6], particularly among obese individuals [4,7].

The present study was initiated hoping to shed some more new light on the role of BNP in the pathophysiology of hypertension and was based on two hypotheses: higher serum concentrations of NT-proBNP, used as a surrogate marker for active BNP, would be associated with prevalent hypertension, whereas lower concentrations would be associated with incident hypertension, indicating that lower BNP concentrations could be causally related to the development of hypertension, whereas higher concentrations would be the consequence of increased left atrial size, left ventricular hypertrophy, and myocardial stretch caused by already developed hypertension with target organ damage.

## Materials and Methods

### Study Population

The present investigation utilized data from the Inter99 study carried out at the Research Center for Prevention and Health in the Capital Region of Denmark. A detailed description of the Inter99 study design and methods, including information on factors determining response to participation, is available elsewhere [8,9]. Briefly, the Inter99 study is a randomized non-pharmacological intervention study for prevention of ischemic heart disease (CT00289237, ClinicalTrials.gov). The Inter99 study was initiated in 1999 and the cohort consists of all 61,301 individuals born in 1939–40, 1944–45, 1949–50, 1954–55, 1959–60, 1964–65 and 1969–70 and living in 11 municipalities in the former Copenhagen County. From this population, an age- and sex-stratified random sample comprising 13,016 individuals was drawn and invited for the intervention. In total, 6,784 (52%) individuals attended the program.

**Fig 1:**
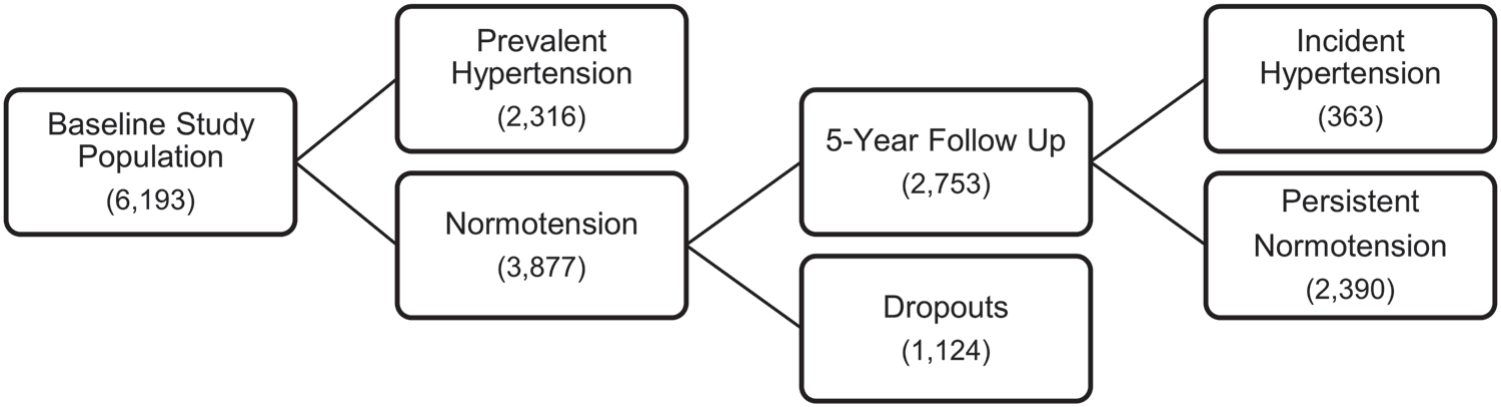
Study flowchart. The figure shows flowchart for this substudy within the Inter99 Study.

Eligible criteria for this study were a full set of blood pressure measurements, data about use of antihypertensive medication, and available NT-proBNP measurements (n = 6,450). One participant did not fulfill the blood pressure measurement criterion, 19 participants did not fulfill the antihypertensive medication criterion, and 314 participants did not have measurements of NT-proBNP. Exclusion criteria were self-reported ischemic heart disease (n = 48), other known cardiovascular diseases (n = 162), and stroke (n = 47), leaving 6,193 participants for the cross-sectional prevalence study. Of these, 2,316 individuals had prevalent hypertension (see definition below), leaving 3,877 potential individuals for further incidence analysis using data from the pre-planned 5-year follow-up. However, after a mean follow-up time of 5.4 years, only 2,753 individuals attended the follow-up examination, where 363 individuals (41 cases per 1,000 subject-years of follow-up) had developed hypertension (Fig. 1). All participants gave a written informed consent before taking part in the study. The Inter99 study was approved by the local ethics committee of Copenhagen County (KA98 155) and the Danish Data Protection Agency. The Inter99 study was conducted in accordance with the Declaration of Helsinki.

### Main Outcome of Interest: Hypertension

At both baseline and at the 5-year follow-up visit, all participants had their blood pressure measured twice with a mercury sphygmomanometer (Mercuro 300, Speidel & Keller) with appropriate cuff size after 5 minutes of rest in the supine position. Trained staff performed all measurements. If systolic blood pressure (SBP) ≥140 mm Hg or diastolic blood pressure (DBP) ≥90 mm Hg, the measurements were repeated twice to minimize the “white coat” effect with the two lowest values being recorded and the average of the recorded measurements was used. Prevalent hypertension was defined as use of antihypertensive medication or SBP ≥140 mm Hg or DBP ≥90 mm Hg at baseline. Incident hypertension was defined a normotension at baseline and use of antihypertensive medication or SBP ≥140 mm Hg or DBP ≥90 mm Hg at the 5-year follow-up visit.

### Exposure Measurements

The invitation included a validated questionnaire to be completed in advance in which information on lifestyle, education, working conditions, chronic diseases among other details were recorded [10,11]. Educational level was classified as none, low, medium, high or missing value, including students. Alcohol intake was recorded as number of standard drinks (12 gr alcohol) per week. Leisure time physical activity was categorized as sedentary, moderate activity, regular exercise and regular hard exercise (the last two categories were merged to high). Based on their dietary habits, participants were classified in three groups: healthy, normal, unhealthy. Smoking habits was recorded as never, former or current.

Height was measured without shoes to the nearest centimeter (cm), weight without shoes and overcoat to the nearest 0.1 kilogram (kg). Body mass index (BMI) was calculated as weight in kg divided by height in meters (m) squared. Overweight was defined as BMI ≥25 kg/m^2^ and obesity as BMI ≥30 kg/m^2^. Waist circumference was measured midway between the lower rib margin and the iliac crest to the nearest cm. Heart rate (beats/min) was derived from an electrocardiogram and forced expiratory volume in one second (FEV1) were recorded as described elsewhere [12].

### Blood Analyses

Fasting blood samples were drawn in the morning and on a daily basis transferred to the laboratory at Steno Diabetes Centre, Gentofte, for analysis. Total cholesterol and triglycerides were determined with enzymatic techniques (Boeringer Mannheim, Germany). Serum insulin was measured using flourimmunoassay technique. HbA1c was measured using the high-performance liquid chromatography technique. The degree of insulin resistance was estimated using the homeostasis model of insulin resistance (HOMA-IR) [13].

Serum concentrations of NT-proBNP were measured using Chemilumineszenz on platform Dimension VISTA (Siemens Healthcare Diagnostics, Eschborn, Germany) with coefficients of variation of 8.90%, 6.89%, and 9.68% for low, medium and high serum concentrations, respectively. Serum creatinine was also measured on platform Dimension VISTA using an enzymatic technique, and glomerular filtration rate (eGFR) was estimated using the Chronic Kidney Disease Epidemiology Collaboration (CKD-EPI) equation [14]. Serum concentrations of leptin were analyzed at Tethys Bioscience, Emeryville, California, using an ultrasensitive molecular counting technology platform (Singulex, St. Louis, MO, USA). Reagents were obtained from R & D Systems (Minneapolis, MN, USA) and from U.S. Biological (Swampscott, MA, USA). Leptin concentrations were calculated as the mean of three replicates. Assays had dynamic ranges of 10^2^–10^3^, intraplate coefficients of variation of ≤5% and an average lower limit of detection of 10 pg/mL.

### Statistical Analyses

All analyses were performed with SAS, version 9.3 (SAS Institute, Cary, NC, USA). Baseline descriptive data (shown in Table 1), expressed as the mean ± standard deviation (SD) or as median and 5^th^ to 95^th^ percentile depending on distribution or proportions in percent, were compared with parametric or non-parametric statistics as appropriate for continuous variables and X^2^ tests as appropriate for categorical variables. Partial Spearman correlation analysis (shown in Table 2), adjusted for age and sex, were used to identify significant relationships between NT-proBNP and variables of interest. Multiple logistic regression models were constructed to compute standardized odds ratios (OR) with 95% confidence intervals (CI) per SD increase in baseline serum NT-proBNP concentrations with both prevalent and incident hypertension as outcome. Variables, which displayed a skewed distribution, were entered in the logistic regression models after logarithmic (log2) transformation. In the various models, missing data were handled with the pairwise deletion technique, which involves deleting a case when it is missing a variable required for a particular analysis, but including that case in analyses for which all required variables are present. Because of this, total n and cases decrease subsequently from model 1 to 4 (as shown in Table 3 and 4). In the multiple logistic regression models, we included variables reported to be associated with NT-proBNP or BNP concentrations in serum [7,15–18], and variables known to be associated with hypertension [19–24] with successive inclusion of sets of variables in the various models. In model 1, we adjusted for age and sex. In model 2, we added adjustments for heart rate, eGFR, BMI, FEV1, and baseline SBP, entered as <120, 120–129, and 130–39 mm Hg, and baseline DBP, entered as <80, 80–84.and 85–89 mm Hg, with incident hypertension as outcome. In model 3, we further included life style risk factors, such as alcohol intake, physical activity, smoking status, educational level, and dietary habits. Finally, in model 4, we added adjustments for metabolic variables, such as HOMA-IR, HbA1c, triglycerides, cholesterol, and leptin. The validity of the multiple logistic regression models was checked by Hosmer and Lemeshow goodness-of-fit test, and regarding the categorization of SBP and DBP, this was done to comply with Hosmer and Lemeshow goodness-of-fit test. We tested model accuracy using C-statistics. In the logistic regression models, the linearity assumption was checked by adding the term squared and cubed to the model and checking for significance. As we found no sex-based differences in the relationship between NT-proBNP and both prevalent and incident hypertension (*P*>0.49 for interaction), we pooled all the data together to increase power in our main outcome analyses. Finally, to compare differences in baseline NT-proBNP concentrations between various subgroups of interest, we used the SAS LSMEANS statement to generate adjusted means for NT-proBNP, and because of the skewed distribution of NT-proBNP, these results are presented as percentages. All statistical tests were two-sided and the significance level was chosen as *P*<0.05.

**Table 1:** Baseline characteristics of the study population stratified by hypertension status.

Variables	Normotension	Hypertension
Number of participants	3,877	2,316
Demographic		
Female sex, %	57.8	41.3†
Age, years	44.1±8	49.0±7†
Self-reported diabetes, %	1.2	3.0†
Parental history of hypertension, %	36.0	43.8†
High educational level, %	30.3	26.5*
Use of antihypertensive drugs, %	0	15.9†
Life style		
Alcohol intake, standard drinks/week	6 (0–28)	8 (0–41) †
High physical activity, %	40.4	40.5
Physical activity, hours/week	4.5±1.2	4.5±1.3
Unhealthy dietary habits, %	15.1	16.2
Smoking, %		
Never	33.4	38.3†
Former	22.9	29.4†
Current	43.7	32.3†
Anthropometric		
Body mass index, kg/m^2^	25.2±4	28.1±5†
Waist circumference, cm	83±12	92±13†
Hemodynamic and pulmonic		
Heart rate, beats/min	65±10	70±12†
Systolic blood pressure, mm Hg	120±10	146±15†
Diastolic blood pressure, mm Hg	76±7	93±9†
FEV1, litre	3.2±0.8	3.2±0.8
Lipid		
Total cholesterol, mmol/L	5.3±1.1	5.8±1.1†
HDL-Cholesterol, mmol/L	1.5±0.4	1.4±0.4†
Triglycerides, mmol/L	1.0(0.5–2.4)	1.3(0.6–3.6) †
Metabolic and renal		
Leptin, ng/mL	5.5(0.8–30.5)	6.2(1.1–38.7) †
Insulin, pmol/L	30(12–84)	41(16–117) †
Haemoglobin A1c, %	5.8±0.5	6.0±0.8†
HOMA-IR, units	1.2(0.5–3.6)	1.7(0.6–5.6) †
NT-proBNP—males, pg/mL	27(9–94)	30(8–124) †
NT-proBNP—females, pg/mL	55(18–166)	61(19–224) †
Creatinine, mg/dL	72±14	74±15†
Estimated GFR, ml/min/1.73m^2^	92.2±16.6	84.8±14.9†

Data are presented as mean ±standard deviation for normally distributed variables, as median (5^th^ to 95^th^ percentile) for skewed distributed variables, and frequency in percent for categorical variables.

FEV1 indicates forced expiratory volume in one sec.; HDL, High-density lipoprotein; HOMA, homeostatic model assessment; IR, insulin resistance; NT-proBNP, N-terminal pro-B-type natriuretic peptide; GFR, glomerular filtration rate.

*P<0.*01*

†*P<0*.*001*

**Table 2:** Partial Spearman’s rank correlation coefficients, adjusted for sex and age, between serum N-terminal pro-B-type natriuretic peptide and selected variables of interest.

Variables	Prevalent Study Population	Incident Study Population
Anthropometric		
Body mass index, kg/m^2^	-0.08‡	-0.05*
Waist circumference, cm	-0.09‡	-0.05†
Hemodynamic and pulmonic		
Heart rate, beats/min	-0.07‡	-0.07‡
Systolic blood pressure, mm Hg	0.04†	0.00
Diastolic blood pressure, mm Hg	-0.02	-0.06‡
FEV1, litre	-0.02	-0.03
Lipid		
Total cholesterol, mmol/L	-0.16‡	-0.15‡
HDL-Cholesterol, mmol/L	0.06‡	0.03
Triglycerides, mmol/L	-0.10‡	-0.06†
Metabolic, inflammatory and renal		
Leptin, ng/mL	-0.07‡	-0.05*
Insulin, pmol/L	-0.13‡	-0.08‡
Haemoglobin A1c, %	-0.06‡	-0.02
HOMA-IR, units	-0.13‡	-0.09‡
Estimated GFR, ml/min/1.73m^2^	-0.01	-0.03

FEV1 indicates forced expiratory volume in one sec.; HDL, High-density lipoprotein; HOMA, homeostatic model assessment; IR, insulin resistance; GFR, glomerular filtration rate.

*P<0.*05*

†*P<0*.*01*

‡*P<0*.*001*

**Table 3:** Multivariate analysis of the association of serum N-terminal pro-B-type natriuretic peptide and prevalent hypertension.

	At risk	Cases	Odds Ratio per 1 SD Increment in Log NT-proBNP Values (95%CI)	P Value
Model 1	6,193	2,316	1.08 (1.02 to 1.14)	*0.011*
Model 2	5,769	2,161	1.16 (1.09 to 1.23)	*<0.001*
Model 3	5,648	2,111	1.17 (1.10 to 1.24)	*<0.001*
Model 4	5,307	1,979	1.21 (1.13 to 1.30)	*<0.001*

**Model 1:** adjusted for age and sex.

**Model 2:** adjusted for model 1, heart rate, estimated glomerular filtration rate, body mass index, and FEV1.

**Model 3:** adjusted for model 2, alcohol intake, physical activity, smoking status, educational level, and dietary habits.

**Model 4:** adjusted for model 3, homeostatic model assessment of insulin resistance, haemoglobin A1c, triglycerides, total cholesterol, and leptin.

**Table 4:** Multivariate analysis of the association of serum N-terminal pro-B-type natriuretic peptide and incident hypertension.

	At risk	Cases	Odds Ratio per 1 SD Increment in Log NT-proBNP Values (95%CI)	*P Value*
Model 1	2,753	363	0.89 (0.79 to 1.00)	*0.048*
Model 2	2,556	344	0.89 (0.79 to 1.00)	*0.047*
Model 3	2,525	339	0.88 (0.78 to 0.99)	*0.039*
Model 4	2,389	324	0.86 (0.76 to 0.98)	*0.020*

**Model 1:** adjusted for age and sex.

**Model 2:** adjusted for model 1, heart rate, estimated glomerular filtration rate, body mass index, FEV1, baseline systolic- and diastolic blood pressure (three categories each).

**Model 3:** adjusted for model 2, alcohol intake, physical activity, smoking status, educational level, and dietary habits.

**Model 4:** adjusted for model 3, homeostatic model assessment of insulin resistance, haemoglobin A1c, triglycerides, total cholesterol, and leptin.

## Results

Some general characteristics of the study population stratified by hypertension status at the baseline examination in 1999 are summarized in Table 1. Participants with hypertension were older and more men than women had hypertension (*P*<0.001). Furthermore, participants with hypertension had a higher proportion with a positive family history of hypertension, had more often a low education level (*P*<0.01), a higher alcohol intake, a higher BMI, a lower eGFR and were more dyslipidemic and dysmetabolic and had higher serum concentrations of NT-proBNP (*P*<0.001).

The results of our partial Spearman correlation analyses, adjusted for age and sex, are shown in Table 2. We studied the relationships between NT-proBNP and variables of interest in the entire study population examined in 1999—named “the prevalent study population”—and in the participants who were normotensive in 1999 and showed up for the follow-up examination 5 years later—named “the incident study population”. Significant negative relationships were found between NT-proBNP and anthropometric and lipid variables and serum insulin. In addition, NT-proBNP was significantly related to SBP (*P*<0.01) in the prevalent study population, whereas in the incident population NT-proBNP was significantly negatively related to DBP (*P*<0.001).

**Fig 2:**
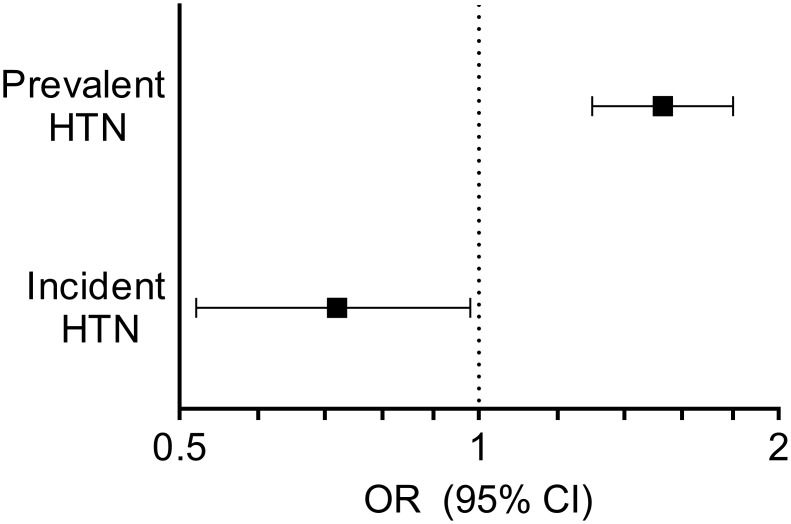
Odds ratios for hypertension according to N-terminal pro-B-type natriuretic peptide concentrations. The figure shows odds ratios with 95% confident intervals for prevalent and incident hypertension for participants with baseline serum N-terminal pro-B-type natriuretic peptide concentrations above the 80^th^ percentile versus participants with baseline N-terminal pro-B-type natriuretic peptide concentrations below the 80^th^ percentile in the fully adjusted models (model 4 in Table 3 and Table 4). The odds ratio estimates with 95% confidence intervals are depicted along the x-axis. HTN indicates hypertension. OR indicates odds ratio.

Table 3 shows the associations between serum concentrations of NT-proBNP and prevalent hypertension based on logistic regression analyses. Adjusted for age and sex, the odds of prevalent hypertension increased 8 percent per SD increase in log-transformed serum concentrations of NT-proBNP (*P* = 0.011). Further adjustments in model 2 to 4 only increased the strength of the association up to 21 percent higher odds of prevalent hypertension per SD increase in NT-proBNP. In the fully adjusted model 4, NT-proBNP concentrations were 12.2% (95%CI 8.2 to 16.1, *P*<0.001) higher among individuals with prevalent hypertension compared with normotensive individuals. C-statistics for model 4 with prevalent hypertension as outcome was 0.798. Furthermore, the odds ratio of prevalent hypertension was 1.53 (95%CI 1.30 to 1.80, *P<0*.*001*) when we compared participants with NT-proBNP values above the 80^th^ percentile with those below the 80^th^ percentile in the fully adjusted model 4 (Fig. 2). Finally, we found no interactions between NT-proBNP and age and BMI with prevalent hypertension as outcome (*P>*0.32*)*.

Table 4 shows the associations between serum concentrations of NT-proBNP and incident hypertension based on logistic regression analyses. Of the 363 participants who had developed incident hypertension, 80 qualified for this diagnosis because they had started antihypertensive medication. Adjusted for age and sex, the odds of incident hypertension decreased 11 percent per SD increase in log-transformed serum concentrations of NT-proBNP. After further adjustments, model 2 to 4, the association between NT-proBNP and incident hypertension was still significant. In contrast to the prevalent analysis, in the fully adjusted model, NT-proBNP concentrations were 10.0% (95%CI 1.1 to 19.8, *P* = 0.028) lower at baseline among individuals who developed hypertension compared with persistently normotensive individuals. C-statistics for model 4 with incident hypertension as outcome was 0.745. The odds ratio of incident hypertension was 0.72 (95%CI 0.53 to 0.99, *P* = 0.046) when we compared participants with NT-proBNP values above the 80^th^ percentile with those below the 80^th^ percentile in the fully adjusted model 4 (Fig. 2). Finally, we found no significant interactions between NT-proBNP and age and baseline blood pressure and BMI with incident hypertension as outcome (*P*>0.29)

Although we did not find any significant interaction between NT-proBNP and BMI with incident hypertension as outcome (*P* = 0.86), we performed a stratified analysis dividing the study population into two groups with BMI ≥25 kg/m^2^ (n = 1,271) and <25 kg/m^2^ (n = 1,482), because we anticipated that a low serum concentration of NT-proBNP could after all be more predictive of risk of incident hypertension in the overweight segment of our study population. Overweight participants had lower baseline serum NT-proBNP concentrations (median [5^th^ to 95^th^ percentile]; 36 pg/mL [10 to 144] versus 45 pg/mL [12 to 148], *P*<0.001) despite higher SBP (mean ±SD; 124±10 mm Hg versus 119±10 mm Hg, *P*<0.001) and higher DBP (78±7 mm Hg versus 75±7 mm Hg, *P*<0.001) compared with normal weight participants, and in the fully adjusted model 4, the odds ratio for incident hypertension was 0.82 (95%CI 0.69–0.96, *P* = 0.016) in overweight participants, whereas the corresponding estimate was 0.97 (95% CI 0.78–1.19, *P* = 0.75) in participants with BMI <25 kg/m^2^. Furthermore, we also examined the associations between baseline NT-proBNP and changes in BP in the overweight segment of “the incident study population”, and although not statistically significant (*P* = 0.13), one SD lower NT-proBNP concentration at baseline was associated with a 0.5 mm Hg higher SBP at follow-up taking use of antihypertensive medication into account [25]. Finally, excluding participants with serum concentrations of NT-proBNP exceeding 200 pg/mL (n = 156), indicative of subclinical structural heart disease [26], or applying the NT-proBNP adjustment algorithm, as suggested by Luchner et al. [27], did not change the results in the main analyses (data not shown).

## Discussion

The major new finding of this study was that in generally healthy individuals from the general population, a higher serum concentration of NT-proBNP was associated with a lower risk of incident hypertension. As expected, we also found that higher serum concentrations of NT-proBNP were associated with higher odds of prevalent hypertension.

Although this study is the first prospective study to report that lower serum concentrations of NT-proBNP are associated with an increased risk of hypertension, some cross-sectional studies have reported that BNP or fragments of the BNP prohormone exhibit a biphasic dose-response relationship with blood pressure and various anthropometric and metabolic variables associated with an increased risk of hypertension [19–21,28]. Thus, in a random sample from Rochester, Minnesota, consisting of 2,082 individuals, age above 45 years, participants with prehypertension and stage I hypertension unexpectedly had (or tended to have when taking their higher blood pressure into account) lower circulating concentrations of BNP and NT-proBNP than normotensive participants, whereas participants with stage II hypertension had the highest circulating concentrations of BNP and NT-proBNP [29]. Furthermore, in the Rochester study, atrial natriuretic peptide (ANP) and proANP were also measured, and here circulating ANP and proANP concentrations unexpectedly did not increase with higher stages of hypertension [29]. So, based on these findings, the authors concluded that human hypertension to some degree is characterized by a lack of activation of the antihypertensive cardiac hormones ANP and BNP, particularly in the early stages of the disease [29]. With respect to the biphasic associations between BNP or fragments of proBNP and metabolic and anthropometric variables associated with hypertension [19,21,28], in the Multi-Ethnic Study of Atherosclerosis, including 5,597 individuals, 45–84 years of age, the well-established negative associations between NT-proBNP and BMI and insulin resistance were only found in participants with NT-proBNP concentrations <100 pg/mL (the physiological range), whereas at higher NT-proBNP concentrations (the pathological range), these associations were blunted [30].

Several prospective studies have investigated the association between BNP and incident hypertension. In 2003, Freitag et al. reported that among 1,801 normotensive Framingham Heart Study participants, mean age 56 years, 286 participants had developed hypertension on follow-up 4 years from baseline, but after adjusting for other covariates, plasma BNP was not related to the incidence of hypertension in either women or men, although BNP was reported to be associated with progression of blood pressure by one or more Joint National Committee VI categories on follow-up in men [31]. Furthermore, there was no evidence of effect modification by BMI in either gender [31]. In 2007, Wang et al. reported that among 1,456 nonhypertensive Framingham Heart Study participants, mean age 56 years, 232 participants had developed hypertension over a mean follow-up of 3 years, but plasma BNP was not related to the incidence of hypertension, whereas significant positive associations were found for C-reactive protein, plasminogen activator inhibitor-1, and urinary albumin/creatinine ratio [32]. In the Jackson Heart Study, Fox et al. reported that among 888 Jackson Heart Study participants, mean age 47 years, 171 participant had developed hypertension after a median follow-up of 5 years, but although baseline circulating plasma BNP concentration predicted an increase in systolic blood pressure, diastolic blood pressure, and in the progression of BP category over a 5-year follow-up period, there was no significant relation of plasma BNP concentration to incident hypertension over the follow-up period, and again no interaction between BNP and BMI on incident hypertension was found [33]. Finally, among 5,026 Japanese normotensive participants, mean age 53 years, 1,022 participants had developed hypertension on follow-up examination 5 years from baseline, but plasma BNP did not predict new-onset hypertension after adjusting for other covariates, including baseline blood pressure [34]. The reasons for the discrepancies between the various studies are not entirely clear but could be related to ethnicity (the Jackson Heart Study only included African Americans) [33] and age (the participants in the Framingham Heart Study were older than in our study) [31].

Based on human physiology and pathophysiology, our results and the results from other studies seem to make some sense and could be relevant to our understanding of the pathophysiology of overweight- and obesity-related hypertension, because overweight and obesity in particular are associated with lower circulating concentrations of BNP and fragments of the BNP prohormone, as well as lower circulating concentrations of ANP and fragments of the ANPs prohormone for that matter [4,7,15,16,35]. In this context, it is important to note that although ANP and BNP seem to respond to the many of the same physiological and pathophysiological stimuli [1,5, 6,36] their circulating concentrations show close correlations (age- and sex-adjusted Spearman correlation coefficient of 0.51, *P*<0.001) [7], their individual genetic variations associate with both peptides in human plasma [2], and they, to a large degree, have the same actions [5,6] ANP is released much more acutely than BNP in response to an intravenous saline loading and acute increases in blood pressure [37–39]. Accordingly, although a short-term low-dose infusion of human BNP in healthy volunteers, producing somehow higher than physiologically relevant BNP concentrations, does not appear to lower blood pressure acutely, it does affect renal, neural, and hormonal factors which could lead to lower long-term blood pressure [40–43], as suggested by the genetic studies which have demonstrated that genetically, and thereby chronically, elevated serum concentrations of BNP and ANP or fragments of the BNP and ANP prohormones are associated with lower systolic and diastolic blood pressure and a reduced risk of hypertension [2,3]. Thus, it is reasonable to speculate that lower circulating concentrations of BNP and ANP, caused by overweight and obesity [4], [7,15,16,35], in the long-run could increase blood pressure and eventually cause hypertension through a relative deficiency of these peptides [5,6], resulting in diminished vasodilation and natriuresis. Nevertheless, when the hypertensive condition has become more severe, owing to an increase in total peripheral resistance, through vasoconstriction and chronic volume overload [44], then the concentrations of BNP and ANP will start to increase as a consequence of myocardial stretch and strain caused by hypertension and the complications related to hypertension [1].

The present study must be interpreted within the context of its potential limitations. It is a limitation that we did not measure active BNP, or active ANP for that matter. Nevertheless, because r^2^ values exceeding 0.4 between NT-proBNP and BNP have been reported [45], and because genotypes that are associated with higher circulating concentrations of BNP are associated with higher circulating concentrations of NT-proBNP [2], it seems reasonable to assume that higher serum NT-proBNP concentrations translated into higher serum concentrations of active BNP in the present study. With respect to ANP, it could be perceived as a limitation that we have no measurements of ANP or fragments of the ANP prohormone, because in generally healthy individuals serum concentrations of ANP seem to be relatively more responsive to changes in salt loads and blood pressure than BNP [37–39]. Furthermore, an estimate of the participants’ sodium intake would have been useful in this study of a surrogate marker for the active natriuretic peptide BNP, as it would have been advantageous if we had had echocardiographic measures. Finally, it is a limitation that the drop-out rate from baseline to follow-up was substantial. Nevertheless, drops-outs and participants who stayed in the study had similar baseline serum NT-proBNP concentrations (median (5^th^ to 95^th^ percentile): 42 pg/mL (11–147) vs 41 pg/mL (11–138) P = 0.10).

Strengths of the present investigation include the large sample size with a substantial number of incident cases, standardized ascertainment of many relevant clinical and biochemical covariates, use of multivariable analyses, including extensive control for covariates, and the possibility to study the association between NT-proBNP and hypertension in a prospective design.

## Conclusions

Based on normal physiology, higher blood pressure leads to higher circulating concentrations of natriuretic peptides [5,6], but based on our data and data from the medical literature, in particular the genetic studies [2,3], a relative deficiency of the natriuretic peptides, resulting in diminished vasodilation and natriuresis, could be involved in the pathogenesis of hypertension in its early states. Because overweight, the strongest risk factor for hypertension [19,20–23], is associated with lower circulating concentrations of natriuretic peptides [4,7,15,16,35] it is reasonable to speculate that a least part of the overweight-related hypertension is mediated by a lack of activation of the antihypertensive cardiac hormones ANP and BNP in the early stages of the disease, an idea our study also supports.
